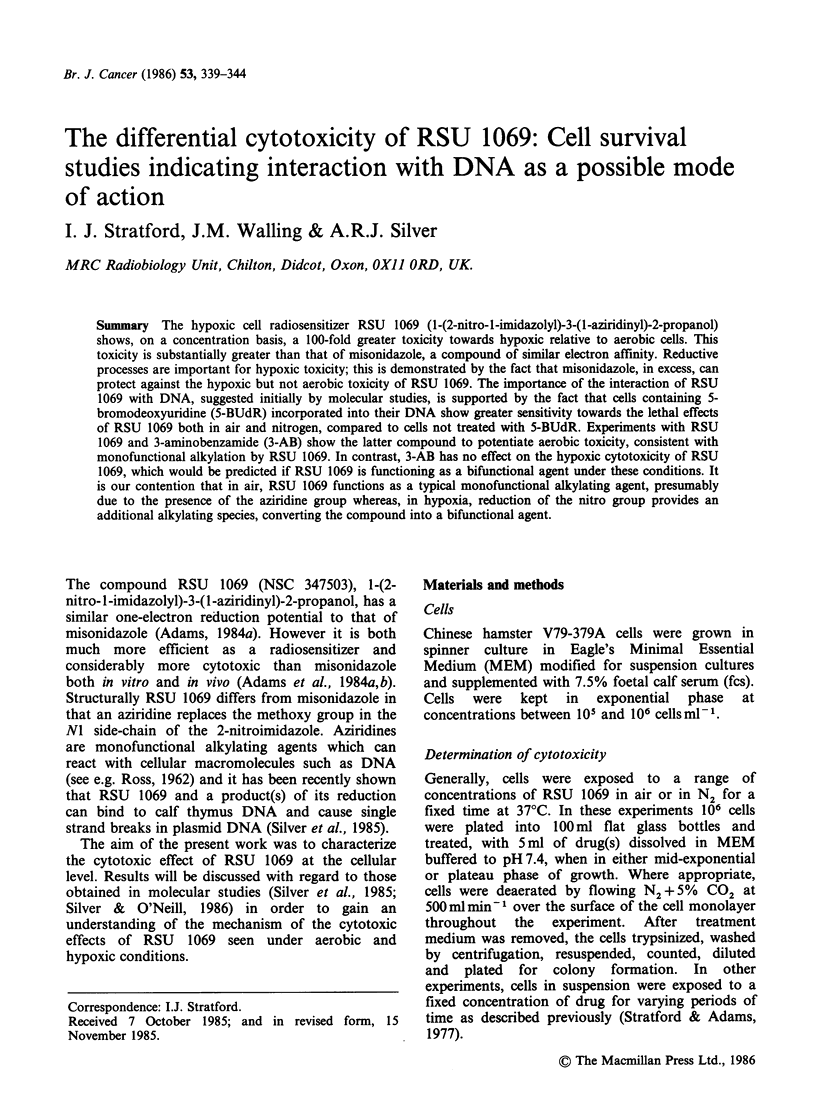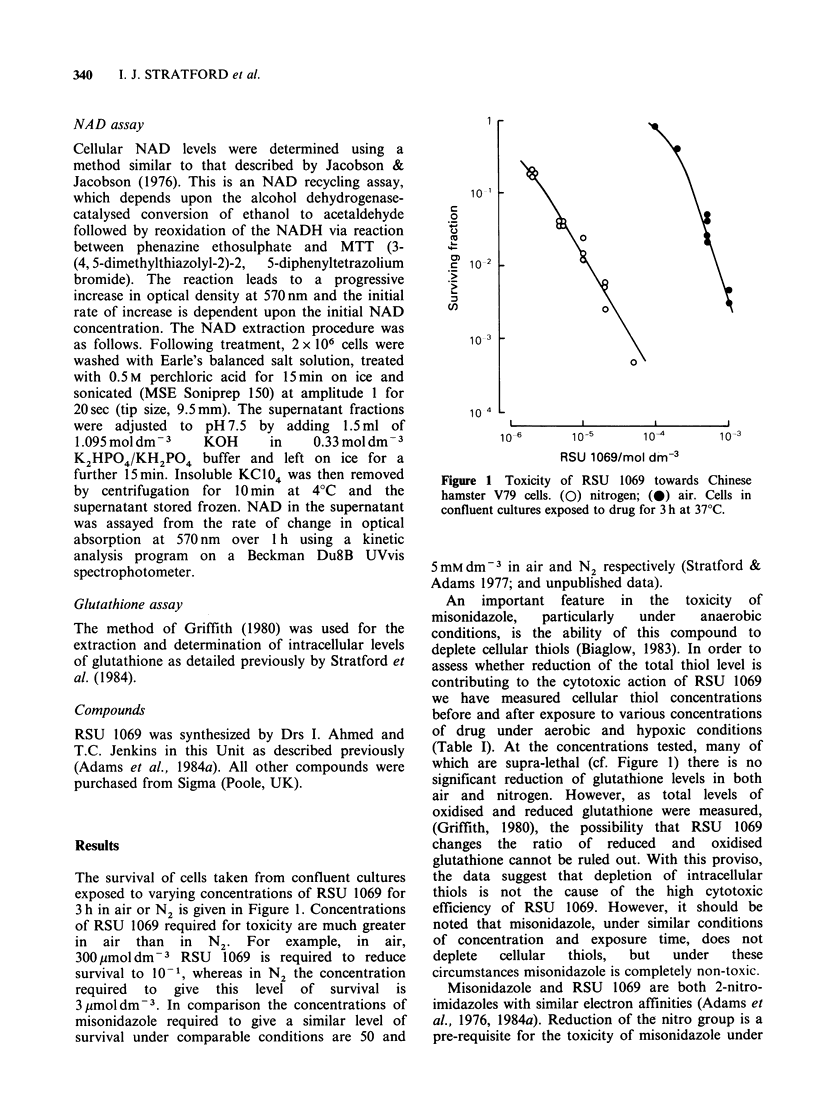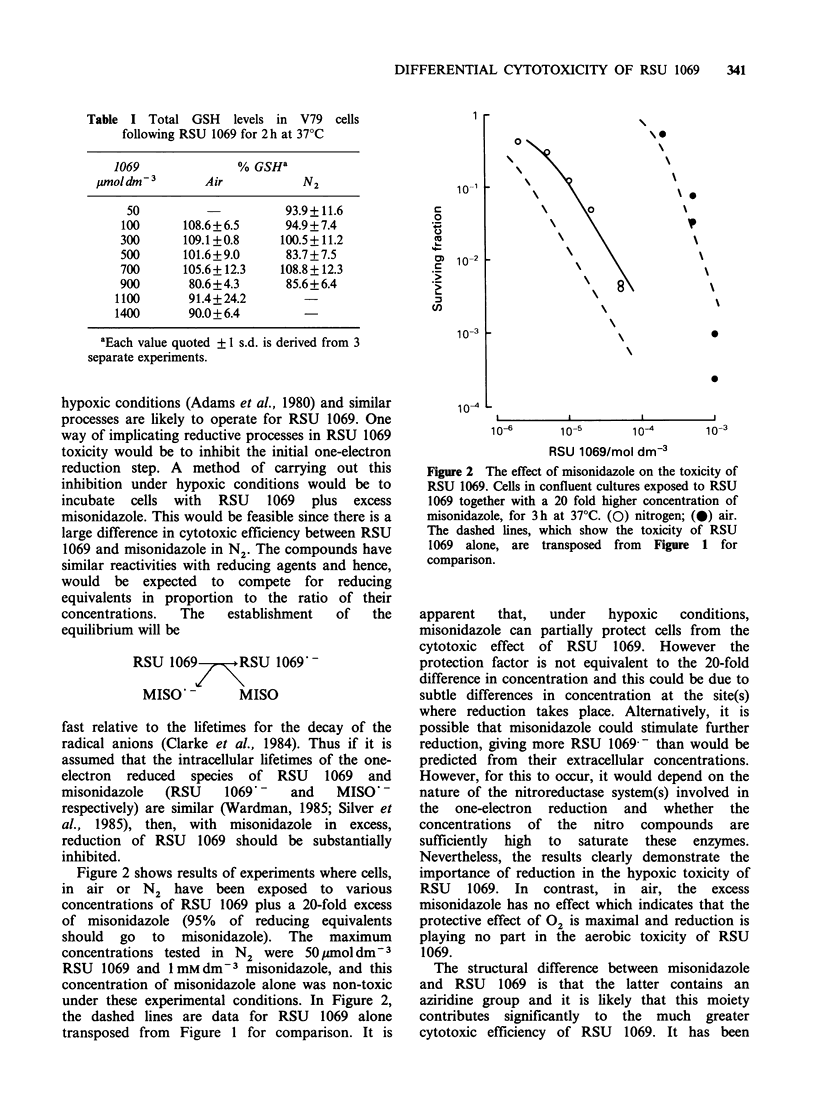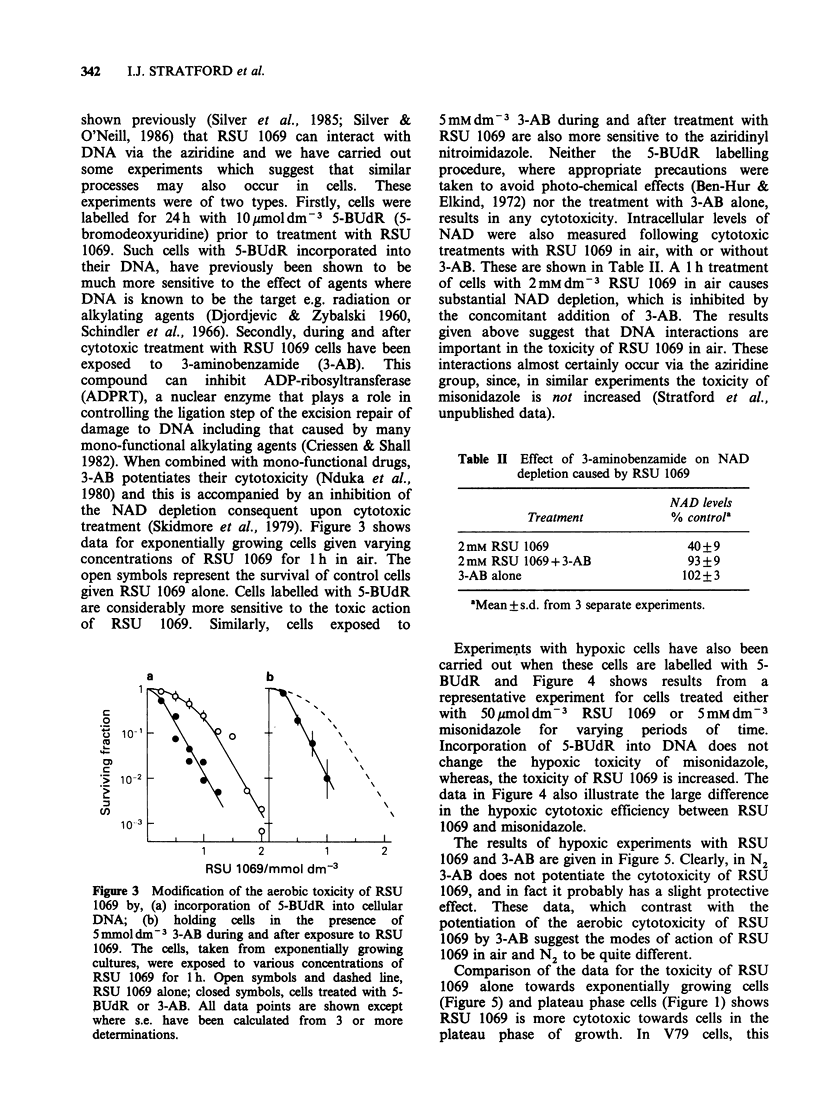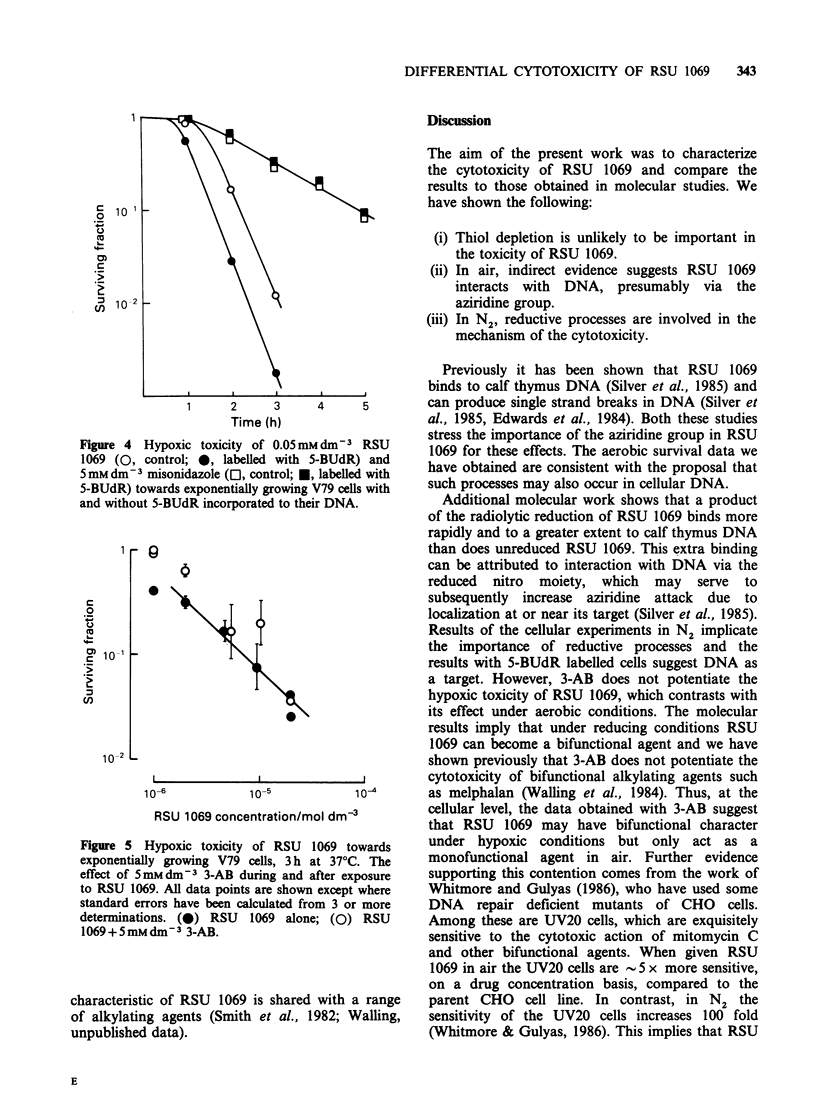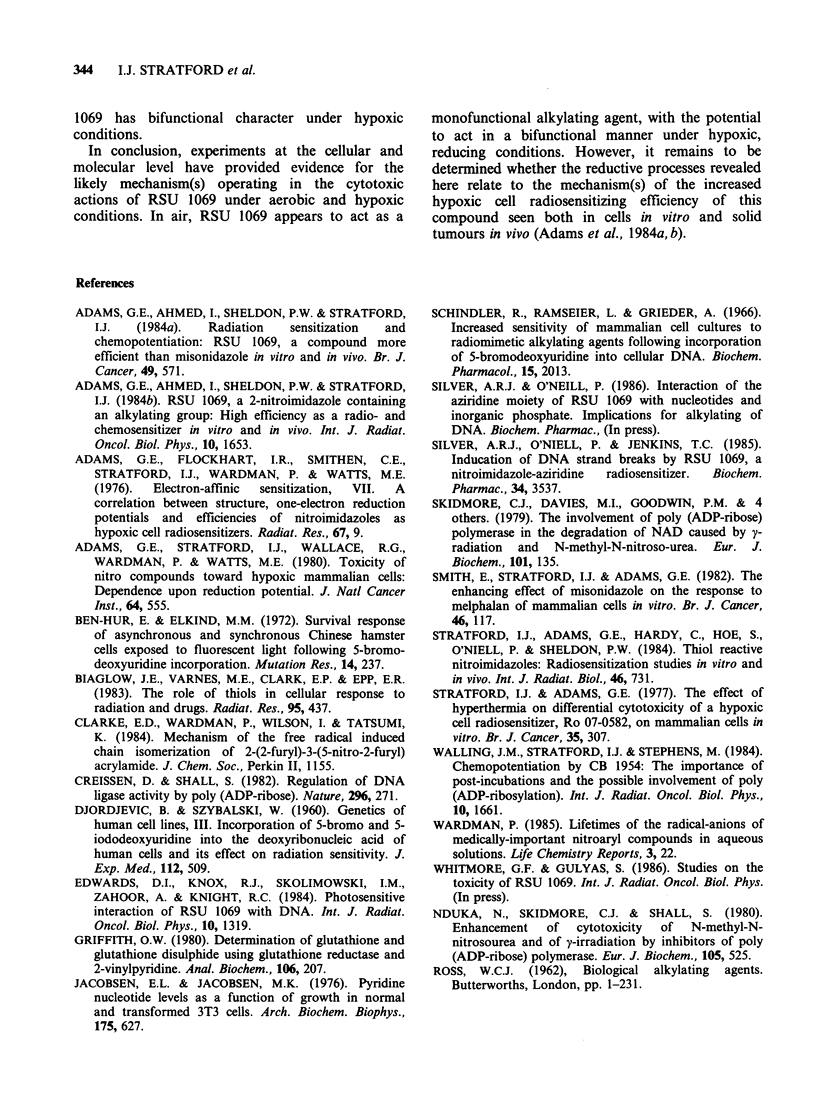# The differential cytotoxicity of RSU 1069: cell survival studies indicating interaction with DNA as a possible mode of action.

**DOI:** 10.1038/bjc.1986.57

**Published:** 1986-03

**Authors:** I. J. Stratford, J. M. Walling, A. R. Silver

## Abstract

The hypoxic cell radiosensitizer RSU 1069 (1-(2-nitro-1-imidazolyl)-3-(1-aziridinyl)-2-propanol) shows, on a concentration basis, a 100-fold greater toxicity towards hypoxic relative to aerobic cells. This toxicity is substantially greater than that of misonidazole, a compound of similar electron affinity. Reductive processes are important for hypoxic toxicity; this is demonstrated by the fact that misonidazole, in excess, can protect against the hypoxic but not aerobic toxicity of RSU 1069. The importance of the interaction of RSU 1069 with DNA, suggested initially by molecular studies, is supported by the fact that cells containing 5-bromodeoxyuridine (5-BUdR) incorporated into their DNA show greater sensitivity towards the lethal effects of RSU 1069 both in air and nitrogen, compared to cells not treated with 5-BUdR. Experiments with RSU 1069 and 3-aminobenzamide (3-AB) show the latter compound to potentiate aerobic toxicity, consistent with monofunctional alkylation by RSU 1069. In contrast, 3-AB has no effect on the hypoxic cytotoxicity of RSU 1069, which would be predicted if RSU 1069 is functioning as a bifunctional agent under these conditions. It is our contention that in air, RSU 1069 functions as a typical monofunctional alkylating agent, presumably due to the presence of the aziridine group whereas, in hypoxia, reduction of the nitro group provides an additional alkylating species, converting the compound into a bifunctional agent.


*Br. J. Cancer* (1986) **53**, 339–344

### The differential cytotoxicity of RSU 1069: Cell survival studies indicating interaction with DNA as a possible mode of action

I. J. Stratford, J.M. Walling & A.R.J. Silver

*MRC Radiobiology Unit, Chilton, Didcot, Oxon, 0X11 0RD, UK.*

**Summary** The hypoxic cell radiosensitizer RSU 1069 (1-(2-nitro-1-imidazolyl)-3-(1-aziridinyl)-2-propanol) shows, on a concentration basis, a 100-fold greater toxicity towards hypoxic relative to aerobic cells. This toxicity is substantially greater than that of misonidazole, a compound of similar electron affinity. Reductive processes are important for hypoxic toxicity; this is demonstrated by the fact that misonidazole, in excess, can protect against the hypoxic but not aerobic toxicity of RSU 1069. The importance of the interaction of RSU 1069 with DNA, suggested initially by molecular studies, is supported by the fact that cells containing 5-bromodeoxyuridine (5-BUdR) incorporated into their DNA show greater sensitivity towards the lethal effects of RSU 1069 both in air and nitrogen, compared to cells not treated with 5-BUdR. Experiments with RSU 1069 and 3-aminobenzamide (3-AB) show the latter compound to potentiate aerobic toxicity, consistent with monofunctional alkylation by RSU 1069. In contrast, 3-AB has no effect on the hypoxic cytotoxicity of RSU 1069, which would be predicted if RSU 1069 is functioning as a bifunctional agent under these conditions. It is our contention that in air, RSU 1069 functions as a typical monofunctional alkylating agent, presumably due to the presence of the aziridine group whereas, in hypoxia, reduction of the nitro group provides an additional alkylating species, converting the compound into a bifunctional agent.

The compound RSU 1069 (NSC 347503), 1-(2-nitro-1-imidazolyl)-3-(1-aziridinyl)-2-propanol, has a similar one-electron reduction potential to that of misonidazole (Adams, 1984*a*). However it is both much more efficient as a radiosensitizer and considerably more cytotoxic than misonidazole both *in vitro* and *in vivo* (Adams *et al.*, 1984*a,b*). Structurally RSU 1069 differs from misonidazole in that an aziridine replaces the methoxy group in the *N*1 side-chain of the 2-nitroimidazole. Aziridines are monofunctional alkylating agents which can react with cellular macromolecules such as DNA (see e.g. Ross, 1962) and it has been recently shown that RSU 1069 and a product(s) of its reduction can bind to calf thymus DNA and cause single strand breaks in plasmid DNA (Silver *et al.*, 1985).

The aim of the present work was to characterize the cytotoxic effect of RSU 1069 at the cellular level. Results will be discussed with regard to those obtained in molecular studies (Silver *et al.*, 1985; Silver & O'Neill, 1986) in order to gain an understanding of the mechanism of the cytotoxic effects of RSU 1069 seen under aerobic and hypoxic conditions.

Correspondence: I.J. Stratford.
Received 7 October 1985; and in revised form, 15 November 1985.

### Materials and methods

### *Cells*

Chinese hamster V79-379A cells were grown in spinner culture in Eagle's Minimal Essential Medium (MEM) modified for suspension cultures and supplemented with 7.5% foetal calf serum (fcs). Cells were kept in exponential phase at concentrations between $10^5$ and $10^6$ cells $ml^{-1}$.

### *Determination of cytotoxicity*

Generally, cells were exposed to a range of concentrations of RSU 1069 in air or in $N_2$ for a fixed time at 37°C. In these experiments $10^6$ cells were plated into 100 ml flat glass bottles and treated, with 5 ml of drug(s) dissolved in MEM buffered to pH 7.4, when in either mid-exponential or plateau phase of growth. Where appropriate, cells were deaerated by flowing $N_2 + 5\%$ $CO_2$ at 500 ml $min^{-1}$ over the surface of the cell monolayer throughout the experiment. After treatment medium was removed, the cells trypsinized, washed by centrifugation, resuspended, counted, diluted and plated for colony formation. In other experiments, cells in suspension were exposed to a fixed concentration of drug for varying periods of time as described previously (Stratford & Adams, 1977).

© The Macmillan Press Ltd., 1986

*NAD assay*

Cellular NAD levels were determined using a method similar to that described by Jacobson & Jacobson (1976). This is an NAD recycling assay, which depends upon the alcohol dehydrogenase-catalysed conversion of ethanol to acetaldehyde followed by reoxidation of the NADH via reaction between phenazine ethosulphate and MTT (3-(4, 5-dimethylthiazolyl-2)-2, 5-diphenyltetrazolium bromide). The reaction leads to a progressive increase in optical density at 570 nm and the initial rate of increase is dependent upon the initial NAD concentration. The NAD extraction procedure was as follows. Following treatment, $2 \times 10^6$ cells were washed with Earle's balanced salt solution, treated with 0.5 M perchloric acid for 15 min on ice and sonicated (MSE Soniprep 150) at amplitude 1 for 20 sec (tip size, 9.5 mm). The supernatant fractions were adjusted to pH 7.5 by adding 1.5 ml of 1.095 mol dm$^{-3}$ KOH in 0.33 mol dm$^{-3}$ $K_2HPO_4/KH_2PO_4$ buffer and left on ice for a further 15 min. Insoluble $KC10_4$ was then removed by centrifugation for 10 min at 4°C and the supernatant stored frozen. NAD in the supernatant was assayed from the rate of change in optical absorption at 570 nm over 1 h using a kinetic analysis program on a Beckman Du8B UVvis spectrophotometer.

*Glutathione assay*

The method of Griffith (1980) was used for the extraction and determination of intracellular levels of glutathione as detailed previously by Stratford *et al.* (1984).

*Compounds*

RSU 1069 was synthesized by Drs I. Ahmed and T.C. Jenkins in this Unit as described previously (Adams *et al.*, 1984*a*). All other compounds were purchased from Sigma (Poole, UK).

### Results

The survival of cells taken from confluent cultures exposed to varying concentrations of RSU 1069 for 3 h in air or $N_2$ is given in Figure 1. Concentrations of RSU 1069 required for toxicity are much greater in air than in $N_2$. For example, in air, 300 $\mu$mol dm$^{-3}$ RSU 1069 is required to reduce survival to $10^{-1}$, whereas in $N_2$ the concentration required to give this level of survival is 3 $\mu$mol dm$^{-3}$. In comparison the concentrations of misonidazole required to give a similar level of survival under comparable conditions are 50 and

**Figure 1** Toxicity of RSU 1069 towards Chinese hamster V79 cells. (○) nitrogen; (●) air. Cells in confluent cultures exposed to drug for 3 h at 37°C.

5 mM dm$^{-3}$ in air and $N_2$ respectively (Stratford & Adams 1977; and unpublished data).

An important feature in the toxicity of misonidazole, particularly under anaerobic conditions, is the ability of this compound to deplete cellular thiols (Biaglow, 1983). In order to assess whether reduction of the total thiol level is contributing to the cytotoxic action of RSU 1069 we have measured cellular thiol concentrations before and after exposure to various concentrations of drug under aerobic and hypoxic conditions (Table I). At the concentrations tested, many of which are supra-lethal (cf. Figure 1) there is no significant reduction of glutathione levels in both air and nitrogen. However, as total levels of oxidised and reduced glutathione were measured, (Griffith, 1980), the possibility that RSU 1069 changes the ratio of reduced and oxidised glutathione cannot be ruled out. With this proviso, the data suggest that depletion of intracellular thiols is not the cause of the high cytotoxic efficiency of RSU 1069. However, it should be noted that misonidazole, under similar conditions of concentration and exposure time, does not deplete cellular thiols, but under these circumstances misonidazole is completely non-toxic.

Misonidazole and RSU 1069 are both 2-nitroimidazoles with similar electron affinities (Adams *et al.*, 1976, 1984*a*). Reduction of the nitro group is a pre-requisite for the toxicity of misonidazole under

**Table I** Total GSH levels in V79 cells following RSU 1069 for 2 h at 37°C

| *1069* *μmol dm$^{-3}$* | *% GSH*[a] *Air* | *$N_2$* |
|---|---|---|
| 50 | — | 93.9±11.6 |
| 100 | 108.6±6.5 | 94.9±7.4 |
| 300 | 109.1±0.8 | 100.5±11.2 |
| 500 | 101.6±9.0 | 83.7±7.5 |
| 700 | 105.6±12.3 | 108.8±12.3 |
| 900 | 80.6±4.3 | 85.6±6.4 |
| 1100 | 91.4±24.2 | — |
| 1400 | 90.0±6.4 | — |

[a]Each value quoted ±1 s.d. is derived from 3 separate experiments.

hypoxic conditions (Adams *et al.*, 1980) and similar processes are likely to operate for RSU 1069. One way of implicating reductive processes in RSU 1069 toxicity would be to inhibit the initial one-electron reduction step. A method of carrying out this inhibition under hypoxic conditions would be to incubate cells with RSU 1069 plus excess misonidazole. This would be feasible since there is a large difference in cytotoxic efficiency between RSU 1069 and misonidazole in $N_2$. The compounds have similar reactivities with reducing agents and hence, would be expected to compete for reducing equivalents in proportion to the ratio of their concentrations. The establishment of the equilibrium will be fast relative to the lifetimes for the decay of the radical anions (Clarke *et al.*, 1984). Thus if it is assumed that the intracellular lifetimes of the one-electron reduced species of RSU 1069 and misonidazole (RSU 1069˙⁻ and MISO˙⁻ respectively) are similar (Wardman, 1985; Silver *et al.*, 1985), then, with misonidazole in excess, reduction of RSU 1069 should be substantially inhibited.

Figure 2 shows results of experiments where cells, in air or $N_2$ have been exposed to various concentrations of RSU 1069 plus a 20-fold excess of misonidazole (95% of reducing equivalents should go to misonidazole). The maximum concentrations tested in $N_2$ were 50 μmol dm$^{-3}$ RSU 1069 and 1 mM dm$^{-3}$ misonidazole, and this concentration of misonidazole alone was non-toxic under these experimental conditions. In Figure 2, the dashed lines are data for RSU 1069 alone transposed from Figure 1 for comparison. It is

**Figure 2** The effect of misonidazole on the toxicity of RSU 1069. Cells in confluent cultures exposed to RSU 1069 together with a 20 fold higher concentration of misonidazole, for 3 h at 37°C. (○) nitrogen; (●) air. The dashed lines, which show the toxicity of RSU 1069 alone, are transposed from **Figure 1** for comparison.

apparent that, under hypoxic conditions, misonidazole can partially protect cells from the cytotoxic effect of RSU 1069. However the protection factor is not equivalent to the 20-fold difference in concentration and this could be due to subtle differences in concentration at the site(s) where reduction takes place. Alternatively, it is possible that misonidazole could stimulate further reduction, giving more RSU 1069˙⁻ than would be predicted from their extracellular concentrations. However, for this to occur, it would depend on the nature of the nitroreductase system(s) involved in the one-electron reduction and whether the concentrations of the nitro compounds are sufficiently high to saturate these enzymes. Nevertheless, the results clearly demonstrate the importance of reduction in the hypoxic toxicity of RSU 1069. In contrast, in air, the excess misonidazole has no effect which indicates that the protective effect of $O_2$ is maximal and reduction is playing no part in the aerobic toxicity of RSU 1069.

The structural difference between misonidazole and RSU 1069 is that the latter contains an aziridine group and it is likely that this moiety contributes significantly to the much greater cytotoxic efficiency of RSU 1069. It has been shown previously (Silver *et al.*, 1985; Silver & O'Neill, 1986) that RSU 1069 can interact with DNA via the aziridine and we have carried out some experiments which suggest that similar processes may also occur in cells. These experiments were of two types. Firstly, cells were labelled for 24 h with 10 $\mu$mol dm$^{-3}$ 5-BUdR (5-bromodeoxyuridine) prior to treatment with RSU 1069. Such cells with 5-BUdR incorporated into their DNA, have previously been shown to be much more sensitive to the effect of agents where DNA is known to be the target e.g. radiation or alkylating agents (Djordjevic & Zybalski 1960, Schindler *et al.*, 1966). Secondly, during and after cytotoxic treatment with RSU 1069 cells have been exposed to 3-aminobenzamide (3-AB). This compound can inhibit ADP-ribosyltransferase (ADPRT), a nuclear enzyme that plays a role in controlling the ligation step of the excision repair of damage to DNA including that caused by many mono-functional alkylating agents (Criessen & Shall 1982). When combined with mono-functional drugs, 3-AB potentiates their cytotoxicity (Nduka *et al.*, 1980) and this is accompanied by an inhibition of the NAD depletion consequent upon cytotoxic treatment (Skidmore *et al.*, 1979). Figure 3 shows data for exponentially growing cells given varying concentrations of RSU 1069 for 1 h in air. The open symbols represent the survival of control cells given RSU 1069 alone. Cells labelled with 5-BUdR are considerably more sensitive to the toxic action of RSU 1069. Similarly, cells exposed to 5 mM dm$^{-3}$ 3-AB during and after treatment with RSU 1069 are also more sensitive to the aziridinyl nitroimidazole. Neither the 5-BUdR labelling procedure, where appropriate precautions were taken to avoid photo-chemical effects (Ben-Hur & Elkind, 1972) nor the treatment with 3-AB alone, results in any cytotoxicity. Intracellular levels of NAD were also measured following cytotoxic treatments with RSU 1069 in air, with or without 3-AB. These are shown in Table II. A 1 h treatment of cells with 2 mM dm$^{-3}$ RSU 1069 in air causes substantial NAD depletion, which is inhibited by the concomitant addition of 3-AB. The results given above suggest that DNA interactions are important in the toxicity of RSU 1069 in air. These interactions almost certainly occur via the aziridine group, since, in similar experiments the toxicity of misonidazole is *not* increased (Stratford *et al.*, unpublished data).

**Figure 3** Modification of the aerobic toxicity of RSU 1069 by, (a) incorporation of 5-BUdR into cellular DNA; (b) holding cells in the presence of 5 mmol dm$^{-3}$ 3-AB during and after exposure to RSU 1069. The cells, taken from exponentially growing cultures, were exposed to various concentrations of RSU 1069 for 1 h. Open symbols and dashed line, RSU 1069 alone; closed symbols, cells treated with 5-BUdR or 3-AB. All data points are shown except where s.e. have been calculated from 3 or more determinations.

**Table II** Effect of 3-aminobenzamide on NAD depletion caused by RSU 1069

| *Treatment* | *NAD levels % control*[a] |
|---|---|
| 2 mM RSU 1069 | 40 ± 9 |
| 2 mM RSU 1069 + 3-AB | 93 ± 9 |
| 3-AB alone | 102 ± 3 |

[a]Mean ± s.d. from 3 separate experiments.

Experiments with hypoxic cells have also been carried out when these cells are labelled with 5-BUdR and Figure 4 shows results from a representative experiment for cells treated either with 50 $\mu$mol dm$^{-3}$ RSU 1069 or 5 mM dm$^{-3}$ misonidazole for varying periods of time. Incorporation of 5-BUdR into DNA does not change the hypoxic toxicity of misonidazole, whereas, the toxicity of RSU 1069 is increased. The data in Figure 4 also illustrate the large difference in the hypoxic cytotoxic efficiency between RSU 1069 and misonidazole.

The results of hypoxic experiments with RSU 1069 and 3-AB are given in Figure 5. Clearly, in $N_2$ 3-AB does not potentiate the cytotoxicity of RSU 1069, and in fact it probably has a slight protective effect. These data, which contrast with the potentiation of the aerobic cytotoxicity of RSU 1069 by 3-AB suggest the modes of action of RSU 1069 in air and $N_2$ to be quite different.

Comparison of the data for the toxicity of RSU 1069 alone towards exponentially growing cells (Figure 5) and plateau phase cells (Figure 1) shows RSU 1069 is more cytotoxic towards cells in the plateau phase of growth. In V79 cells, this

**Figure 4** Hypoxic toxicity of 0.05 mM dm$^{-3}$ RSU 1069 (○, control; ●, labelled with 5-BUdR) and 5 mM dm$^{-3}$ misonidazole (□, control; ■, labelled with 5-BUdR) towards exponentially growing V79 cells with and without 5-BUdR incorporated to their DNA.

**Figure 5** Hypoxic toxicity of RSU 1069 towards exponentially growing V79 cells, 3 h at 37°C. The effect of 5 mM dm$^{-3}$ 3-AB during and after exposure to RSU 1069. All data points are shown except where standard errors have been calculated from 3 or more determinations. (●) RSU 1069 alone; (○) RSU 1069 + 5 mM dm$^{-3}$ 3-AB.

characteristic of RSU 1069 is shared with a range of alkylating agents (Smith *et al.*, 1982; Walling, unpublished data).

### Discussion

The aim of the present work was to characterize the cytotoxicity of RSU 1069 and compare the results to those obtained in molecular studies. We have shown the following:

(i) Thiol depletion is unlikely to be important in the toxicity of RSU 1069.
(ii) In air, indirect evidence suggests RSU 1069 interacts with DNA, presumably via the aziridine group.
(iii) In $N_2$, reductive processes are involved in the mechanism of the cytotoxicity.

Previously it has been shown that RSU 1069 binds to calf thymus DNA (Silver *et al.*, 1985) and can produce single strand breaks in DNA (Silver *et al.*, 1985, Edwards *et al.*, 1984). Both these studies stress the importance of the aziridine group in RSU 1069 for these effects. The aerobic survival data we have obtained are consistent with the proposal that such processes may also occur in cellular DNA.

Additional molecular work shows that a product of the radiolytic reduction of RSU 1069 binds more rapidly and to a greater extent to calf thymus DNA than does unreduced RSU 1069. This extra binding can be attributed to interaction with DNA via the reduced nitro moiety, which may serve to subsequently increase aziridine attack due to localization at or near its target (Silver *et al.*, 1985). Results of the cellular experiments in $N_2$ implicate the importance of reductive processes and the results with 5-BUdR labelled cells suggest DNA as a target. However, 3-AB does not potentiate the hypoxic toxicity of RSU 1069, which contrasts with its effect under aerobic conditions. The molecular results imply that under reducing conditions RSU 1069 can become a bifunctional agent and we have shown previously that 3-AB does not potentiate the cytotoxicity of bifunctional alkylating agents such as melphalan (Walling *et al.*, 1984). Thus, at the cellular level, the data obtained with 3-AB suggest that RSU 1069 may have bifunctional character under hypoxic conditions but only act as a monofunctional agent in air. Further evidence supporting this contention comes from the work of Whitmore and Gulyas (1986), who have used some DNA repair deficient mutants of CHO cells. Among these are UV20 cells, which are exquisitely sensitive to the cytotoxic action of mitomycin C and other bifunctional agents. When given RSU 1069 in air the UV20 cells are ~5× more sensitive, on a drug concentration basis, compared to the parent CHO cell line. In contrast, in $N_2$ the sensitivity of the UV20 cells increases 100 fold (Whitmore & Gulyas, 1986). This implies that RSU

1069 has bifunctional character under hypoxic conditions.

In conclusion, experiments at the cellular and molecular level have provided evidence for the likely mechanism(s) operating in the cytotoxic actions of RSU 1069 under aerobic and hypoxic conditions. In air, RSU 1069 appears to act as a monofunctional alkylating agent, with the potential to act in a bifunctional manner under hypoxic, reducing conditions. However, it remains to be determined whether the reductive processes revealed here relate to the mechanism(s) of the increased hypoxic cell radiosensitizing efficiency of this compound seen both in cells *in vitro* and solid tumours *in vivo* (Adams *et al.*, 1984*a*, *b*).

### References

ADAMS, G.E., AHMED, I., SHELDON, P.W. & STRATFORD, I.J. (1984*a*). Radiation sensitization and chemopotentiation: RSU 1069, a compound more efficient than misonidazole *in vitro* and *in vivo*. *Br. J. Cancer*, **49**, 571.

ADAMS, G.E., AHMED, I., SHELDON, P.W. & STRATFORD, I.J. (1984*b*). RSU 1069, a 2-nitroimidazole containing an alkylating group: High efficiency as a radio- and chemosensitizer *in vitro* and *in vivo*. *Int. J. Radiat. Oncol. Biol. Phys.*, **10**, 1653.

ADAMS, G.E., FLOCKHART, I.R., SMITHEN, C.E., STRATFORD, I.J., WARDMAN, P. & WATTS, M.E. (1976). Electron-affinic sensitization, VII. A correlation between structure, one-electron reduction potentials and efficiencies of nitroimidazoles as hypoxic cell radiosensitizers. *Radiat. Res.*, **67**, 9.

ADAMS, G.E., STRATFORD, I.J., WALLACE, R.G., WARDMAN, P. & WATTS, M.E. (1980). Toxicity of nitro compounds toward hypoxic mammalian cells: Dependence upon reduction potential. *J. Natl Cancer Inst.*, **64**, 555.

BEN-HUR, E. & ELKIND, M.M. (1972). Survival response of asynchronous and synchronous Chinese hamster cells exposed to fluorescent light following 5-bromodeoxyuridine incorporation. *Mutation Res.*, **14**, 237.

BIAGLOW, J.E., VARNES, M.E., CLARK, E.P. & EPP, E.R. (1983). The role of thiols in cellular response to radiation and drugs. *Radiat. Res.*, **95**, 437.

CLARKE, E.D., WARDMAN, P., WILSON, I. & TATSUMI, K. (1984). Mechanism of the free radical induced chain isomerization of 2-(2-furyl)-3-(5-nitro-2-furyl) acrylamide. *J. Chem. Soc.*, Perkin II, 1155.

CREISSEN, D. & SHALL, S. (1982). Regulation of DNA ligase activity by poly (ADP-ribose). *Nature*, **296**, 271.

DJORDJEVIC, B. & SZYBALSKI, W. (1960). Genetics of human cell lines, III. Incorporation of 5-bromo and 5-iododeoxyuridine into the deoxyribonucleic acid of human cells and its effect on radiation sensitivity. *J. Exp. Med.*, **112**, 509.

EDWARDS, D.I., KNOX, R.J., SKOLIMOWSKI, I.M., ZAHOOR, A. & KNIGHT, R.C. (1984). Photosensitive interaction of RSU 1069 with DNA. *Int. J. Radiat. Oncol. Biol. Phys.*, **10**, 1319.

GRIFFITH, O.W. (1980). Determination of glutathione and glutathione disulphide using glutathione reductase and 2-vinylpyridine. *Anal. Biochem.*, **106**, 207.

JACOBSEN, E.L. & JACOBSEN, M.K. (1976). Pyridine nucleotide levels as a function of growth in normal and transformed 3T3 cells. *Arch. Biochem. Biophys.*, **175**, 627.

SCHINDLER, R., RAMSEIER, L. & GRIEDER, A. (1966). Increased sensitivity of mammalian cell cultures to radiomimetic alkylating agents following incorporation of 5-bromodeoxyuridine into cellular DNA. *Biochem. Pharmacol.*, **15**, 2013.

SILVER, A.R.J. & O'NEILL, P. (1986). Interaction of the aziridine moiety of RSU 1069 with nucleotides and inorganic phosphate. Implications for alkylating of DNA. *Biochem. Pharmac.*, (In press).

SILVER, A.R.J., O'NIELL, P. & JENKINS, T.C. (1985). Inducation of DNA strand breaks by RSU 1069, a nitroimidazole-aziridine radiosensitizer. *Biochem. Pharmac.*, **34**, 3537.

SKIDMORE, C.J., DAVIES, M.I., GOODWIN, P.M. & 4 others. (1979). The involvement of poly (ADP-ribose) polymerase in the degradation of NAD caused by $\gamma$-radiation and N-methyl-N-nitroso-urea. *Eur. J. Biochem.*, **101**, 135.

SMITH, E., STRATFORD, I.J. & ADAMS, G.E. (1982). The enhancing effect of misonidazole on the response to melphalan of mammalian cells *in vitro*. *Br. J. Cancer*, **46**, 117.

STRATFORD, I.J., ADAMS, G.E., HARDY, C., HOE, S., O'NIELL, P. & SHELDON, P.W. (1984). Thiol reactive nitroimidazoles: Radiosensitization studies *in vitro* and *in vivo*. *Int. J. Radiat. Biol.*, **46**, 731.

STRATFORD, I.J. & ADAMS, G.E. (1977). The effect of hyperthermia on differential cytotoxicity of a hypoxic cell radiosensitizer, Ro 07-0582, on mammalian cells *in vitro*. *Br. J. Cancer*, **35**, 307.

WALLING, J.M., STRATFORD, I.J. & STEPHENS, M. (1984). Chemopotentiation by CB 1954: The importance of post-incubations and the possible involvement of poly (ADP-ribosylation). *Int. J. Radiat. Oncol. Biol. Phys.*, **10**, 1661.

WARDMAN, P. (1985). Lifetimes of the radical-anions of medically-important nitroaryl compounds in aqueous solutions. *Life Chemistry Reports*, **3**, 22.

WHITMORE, G.F. & GULYAS, S. (1986). Studies on the toxicity of RSU 1069. *Int. J. Radiat. Oncol. Biol. Phys.* (In press).

NDUKA, N., SKIDMORE, C.J. & SHALL, S. (1980). Enhancement of cytotoxicity of N-methyl-N-nitrosourea and of $\gamma$-irradiation by inhibitors of poly (ADP-ribose) polymerase. *Eur. J. Biochem.*, **105**, 525.

ROSS, W.C.J. (1962), Biological alkylating agents. Butterworths, London, pp. 1–231.